# Use of Acetic Acid to Partially Replace Lactic Acid for Decontamination against *Escherichia coli* O157:H7 in Fresh Produce and Mechanism of Action

**DOI:** 10.3390/foods10102406

**Published:** 2021-10-11

**Authors:** Jiayi Wang, Yue Lei, Yougui Yu, Lebin Yin, Yangyang Zhang

**Affiliations:** 1 College of Food and Chemical Engineering, Shaoyang University, Shaoyang 422000, China; yufly225@163.com (Y.Y.); yinlebin0451@163.com (L.Y.); zy17680464475@163.com (Y.Z.); 2 Institute of Rice Research, Guizhou Academy of Agricultural, Guiyang 550009, China; leiyue0917@163.com

**Keywords:** acetic acid, decontamination, lactic acid

## Abstract

*Escherichia coli* O157:H7 is frequently detected in ready-to-eat produce and causes serious food-borne diseases. The decontamination efficacy of lactic acid (LA) is clearly established. In this study, LA was mixed with acetic acid (AA) to reduce costs while achieving consistent or better inhibitory effects. Time-kill curves and inoculation experiments using fresh-cut spinach and arugula indicated that 0.8%LA+0.2%AA shows similar antibacterial effects to those of 1%LA. To determine whether 1%LA and 0.8%LA+0.2%AA exert antibacterial effects by similar mechanisms, proteomics analysis was used. The proteins related to macromolecule localization, cellular localization, and protein unfolding were uniquely altered after the treatment with 1%LA, and the proteins related to taxis, response to stress, catabolic process, and the regulation of molecular function were uniquely altered after the treatment with 0.8%LA+0.2%AA. Based on these findings, combined with the results of a network clustering analysis, we speculate that cell membrane damage is greater in response to LA than to 0.8%LA+0.2%AA. This prediction was supported by cell membrane permeability experiments (analyses of protein, nucleotide, ATP, and alkaline phosphatase leakage), which showed that LA causes greater membrane damage than 0.8%LA+0.2%AA. These results provide a theoretical basis for the application of an acid mixture to replace LA for produce decontamination.

## 1. Introduction

Fruits and vegetables are rich in essential vitamins, minerals, and fiber [1]. The FDA recommends the daily intake of three to five different vegetables and two to four different fruits [2]. With the acceleration of daily activities, demand for ready-to-eat fruits and vegetables has increased. However, because they are not cooked at high temperatures, there is a higher risk of diseases caused by foodborne pathogens [3]. *Escherichia coli* O157:H7 is often detected in ready-to-eat foods, particularly in fresh-cut vegetables [4]. According to a report from the United States Centers for Disease Control (CDC), in 2018–2020, there were 539 cases of infection by *Escherichia coli* O157:H7, of which 489 cases were related to leafy greens. The most serious events occurred in 2018, when romaine lettuce from the Yuma growing region caused 210 infections in 36 states, including 96 hospitalizations, 27 patients who developed a type of kidney failure called hemolytic uremic syndrome, and five deaths in Arkansas, California, Minnesota, and New York (CDC: Reports of Selected *E. coli* Outbreak Investigations) [5]. Therefore, decontamination is an effective method to ensure the safety of ready-to-eat vegetables.

For fresh fruits and vegetables, non-thermal technologies have shown antibacterial effects without affecting the quality. For example, bacteriophages, cold plasma, and pulsed light technologies have been effectively applied to fresh produce [6,7]. However, the technical equipment is relatively expensive, and the methods are not suitable for the large-scale decontamination of fresh produce. Chemical decontamination methods benefit from a low cost and moderate efficacy; among the established chemical sanitizers, chlorine-based agents have the lowest cost and moderate efficacy, and the concentration of free chlorine was recommended as 10–200 mg/L [6,8,9]. Recent research on chlorine sanitizers is focused on the prevention of the cross-contamination of the washing water [10,11,12,13]. However, chlorine sanitizers were criticized because they generate carcinogenic byproducts (e.g., trihalomethanes, haloacetic acids, haloketones, and chloropicrin) and form chlorate during fresh produce decontamination [6,14,15].

As another type of sanitizer, most organic acids are listed as generally recognized as safe (GRAS) by the FDA, and some studies have indicated that they have a higher efficacy than that of chlorine [16,17,18]. Lactic acid (LA) is the most widely used GRAS organic acid for fresh produce decontamination. However, lactic acid (LA) is relatively expensive. Among the GRAS organic acids (i.e., citric acid, acetic acid, lactic acid, malic acid, succinic acid, tartaric acid, and propionic acid), acetic acid (AA) has the lowest cost, which is about one-third that of LA [2].

In this study, the use of AA to partially replace LA for decontamination was evaluated. Fresh-cut baby spinach and arugula were selected as the models for the analyses of decontamination against *E. coli* O157:H7. Furthermore, the antibacterial activities of organic acids can be attributed to cellular anion accumulation, which is determined by the proportion of undissociated molecules. Compared with dissociated anions, undissociated acidic molecules have stronger lipophilicity, allowing them to penetrate the microbial cell membrane more easily. After penetration, the higher intracellular pH in the environment will promote acid molecule dissociation, and the dissociated anions will accumulate in the cell and exert toxic effects on DNA, RNA, and ATP synthesis [14,19] and promote acid-sensitive protein denaturation and changes in osmotic pressure [20]. We speculate that the effects of the acid mixture (AM) and LA are mediated by different underlying mechanisms, irrespective of the count reduction, due to the addition of dissociated acetate anions. Therefore, another objective of this work was to evaluate the difference in the mechanism underlying the effects of LA and AM using quantitative proteomic technology.

## 2. Materials and Methods

### 2.1. Time-Kill Curve Analysis

A single colony of *E. coli* O157:H7 (NCTC12900) was inoculated into nutrient broth (Hopebio, Qingdao, China) and cultured overnight at 37 °C. After adjusting the culture to 10^7^–10^8^ CFU/mL, 5 mL was centrifuged at 12,000× *g* for 10 min to obtain the cell pellet, followed by three washing steps using 0.85% NaCl solution. Then, the cells were resuspended in 1 mL of sterilized distilled water and supplemented with 4 mL of sanitizer to obtain the desired sanitizer concentration. The treatment groups were treated with 0.8%LA+0.2%AA, 0.6%LA+0.4%AA, 1%LA, and 1% AA, and the control group was treated with sterilized distilled water. After reaction for 0, 20, 40, 60, and 90 s, 1 mL of the above mixture was mixed with 5 mL of 0.04 M K_2_HPO_4_·3H_2_O to neutralize the sanitizer [21].

After serial dilution, the suspension (0.1 mL) was surface-plated on modified sorbitol MacConkey agar (Hopebio, Qingdao, China) for the quantification of *E. coli* O157:H7.

### 2.2. Sample Preparation

Baby spinach and arugula were purchased from Microgreens (Guangzhou, China). After removing the stem and broken leaves, the remaining parts were rinsed for 30 s to remove dirt. The obtained samples were drained using a manual salad spinner sterilized with 75% ethanol.

### 2.3. Inoculation

The inoculation procedure followed our previously described methods [3,14]. A single colony of *E. coli* O157:H7 was inoculated into nutrient broth and cultured overnight at 37 °C. After adjusting the suspension to 10^9^ CFU/mL, 0.5 or 5 mL was added into two stomacher bags containing 200 mL of sterilized 0.85% NaCl solution. Then, 10 g of sample was added to the bag and massaged for 20 min. The sample was then placed on a sterilized plastic tray in a biosafety cabinet for air drying and stored at 4 °C for 12 h to ensure sufficient bacterial attachment. The resulting samples showed a low inoculation level (10^3^–10^4^ CFU/g) and high inoculation level (10^6^–10^7^ CFU/g).

### 2.4. Decontamination and Microbiological Analysis

Minimizing water consumption and wastewater discharge rates remain challenging in the food industry [8]; thus, a ratio of 1:20 (*w*/*v*) that was sufficient to cover fresh-cut baby spinach and arugula was selected in this study. Inoculated samples were added to the sanitizers (as described in Section 2.1) and disinfected for 1.5 min under shaking at 120 rpm. The samples were then washed with tap water for 15 s to remove the residual sanitizer. The samples were transferred to a polyethylene terephthalate box (18 × 13 × 4 cm), packaged using a polyvinyl chloride cling film (Nan Ya, Taiwan, China), and stored at 4 °C [19]. The samples were analyzed on days 3, 5, and 7 [14]. Fifteen grams of each sample were homogenized with 85 mL of sterilized 0.85% NaCl solution for 1.5 min in a stomacher bag [14]. The microbiological counts were obtained as described in Section 2.1.

### 2.5. Sensory Analysis

Sensory analysis was performed at the end of storage (day 7). Nine trained panelists (ages 24–39 years) from Shijiashike Co. Ltd. (Liaoyang, Liaoning, China) were invited to evaluate sensory color, flavor, and crispness. A 3-point scale method, as described by Wang et al. [14], was used for evaluation: 0 ‘dislike extremely, no characteristic of the product’, 5 ‘neither like nor dislike, acceptability threshold’, and 10 ‘like extremely, very good product characteristics’. The plates containing samples were marked on the bottom and reordered before analysis. During the evaluation, only one person was allowed into the room (equipped with a 40-W white light without windows) and was not allowed to communicate with another person after evaluation. Between each time analysis of the flavor, drinking water was used to rinse the mouth three times, and the next evaluation was performed after 30 s.

### 2.6. Protein, Nucleotide, ATP, and AKP Leakage Analysis

After disinfection (treatment for 20 s) and neutralization, as described in Section 2.1, cells were centrifugated at 12,000× *g* for 10 min. The supernatants were filtered through 0.22 μm filters. The protein and nucleotide concentration in the supernatants was measured by a micro protein assay [22] and at a wavelength of 260 nm [23], respectively. ATP concentration and alkaline phosphatase (AKP) activity in the supernatants was measured using test kit (Jiancheng, Nanjing, China).

### 2.7. Proteomic Analysis

#### 2.7.1. Protein Preparation

After disinfection (treatment for 20 s) and neutralization as described in Section 2.1, the cells were collected from the neutralization fluid using a membrane filter (0.22 µm; Millipore, Darmstadt, Germany). The cells on the membrane were washed off using SDT buffer (4% SDS, 100 mM Tris–HCl, 100 mM DTT, pH 8.0), followed by grinding under liquid nitrogen. The samples were then placed in a 100 °C boiling water bath for 10 min, subjected to ultrasonic treatment for 5 min in an ice bath (25 W for 3 s at intervals of 7 s), added to a 100 °C boiling water bath for 5 min, and centrifuged at 14,000× *g* for 30 min. The samples were filtered in a ultrafiltration tube (0.22 μm), and the protein concentration was quantified using BCA Test Kit (Beyotime, Shanghai, China).

#### 2.7.2. Protein Digestion and Peptide Labeling

Total protein from each sample was digested using filter-aided proteome preparation (FASP) method, as described by Wisniewski et al. [24]. The peptide mixture was labeled using the 10-plex™ Isobaric Mass Tagging Kit (Thermo Scientific, Waltham, MA, USA) according to the manufacturer’s instructions.

#### 2.7.3. Peptide Fractionation

To improve the peptide identification quality, fractionation was performed using the Dionex UltiMate3000 HPLC System (Thermo Fisher, Waltham, MA, USA). The Gemini-NX 4.6 × 150 mm column (3 µm, 110 Å) (00F-4453-E0; Phenomenex, Torrance, CA, USA) was used as the chromatographic column. Elution was performed at a flow rate of 400 μL/min with a gradient of 100% buffer A (10 mM ammonium acetate, pH 10.0) for 5 min, 0–40% buffer B (10 mM ammonium acetate in 90% ACN, pH 10.0) for 20 min, 40–100% buffer B for 7 min, and 0–100% buffer A for 8 min. After fractionation, 40 fractions were subjected to vacuum centrifugation, reconstituted into 10 parts, and freeze-dried. The samples were stored at −80 °C until LC–MS/MS.

#### 2.7.4. LC–MS/MS

After equilibrating the Thermo Scientific separation column (75 μm × 25 cm, 5 μm, 100 Å, C18) with 95% buffer A (0.1% formic acid), the sample was automatically loaded on the Thermo Scientific EASY Trap Column (100 μm × 2 cm, 5 μm, 100 Å, C18) and then separated with a linear gradient: 5–28% buffer B (0.1% formic acid in acetonitrile) for 40 min; 28% to 90% buffer B for 2 min; 90% buffer B for 18 min. Orbitrap-ELite (Thermo Finnigan, San Jose, CA, USA) mass spectrometer was used for the analysis. The detailed mass spectrometry parameters were as follows: detection mode: positive ion; scan range of precursor ions: 350–2000 *m*/*z*; resolution of MS: 60,000 at *m*/*z* 200; AGC target: 1e6; Maximum IT for MS: 10 ms; number of scan ranges: 1; dynamic exclusion: 30 s; most intense signals for MS/MS: top 15; MS2 activation type: HCD; isolation window: 2 *m*/*z*; resolution of MS/MS: 15,000 at *m*/*z* 100; microscans: 1; maximum IT for MS/MS: 100 ms; AGC target: 5e4; normalized collision energy: 35 eV; underfill ratio: 0.1%.

#### 2.7.5. Database Searching and Data Analysis

The raw data obtained as described in Section 2.7.4 were processed using Proteome Discover 2.3. A search for fragmentation spectra was performed using the Mascot search engine embedded in Proteome Discoverer against the uniprot_*Escherichia*_*coli*_O157:H7. fasta database. The search parameters were as follows: Type of search: MS/MS Ion search; Enzyme: Trypsin; Mass Values: Monoisotopic; Max Missed Cleavages: 2; Fixed modifications: Carbamidomethyl (C), TMT 10plex (N-term), TMT 10plex (K); Variable modifications: Oxidation (M); Peptide Mass Tolerance: ±20 ppm; Instrument type: ESI-TRAP; Fragment Mass Tolerance: 0.1 Da; Protein Mass; Unrestricted; Decoy database pattern: Ture; Database: uniprot-*Escherichia*_*coli*_O157:H7.fasta. Peptide identification results were filtered against the standard for a false discovery rate of <1%. Peptide ion peak intensities were collected, and the median peptide ratio was calculated. Then, protein quantification data from each channel were processed by the median normalization method to obtain the final protein quantification results.

### 2.8. Bioinformatics and Statistical Analysis

A fold change of >1.2 and *p* < 0.05 were thresholds for the identification of differentially expressed proteins (DEPs) [25]. A Gene Ontology (GO) enrichment analysis was performed using Blast2GO (https://www.blast2go.com/ (accessed on 1 May 2021)) [26]. Protein–protein interaction networks were analyzed using STRING (http://string-db.org/ (accessed on 10 July 2021)) [27], and the interaction score was set to 0.7.

Data for sensory characteristics, microbial counts, protein, and nucleotide leakage were analyzed using SPSS 22.0. Differences in mean values were analyzed using Duncan’s multiple range tests, and *p*-values of <0.05 were considered significant. All data were expressed as means ± standard deviations. All experiments were independently replicated three times. Three samples were taken on each sampling day and were analyzed in duplicate for a total of six analyses per replicate.

## 3. Results and Discussion

### 3.1. Time-Kill Curves

**Figure 1:**
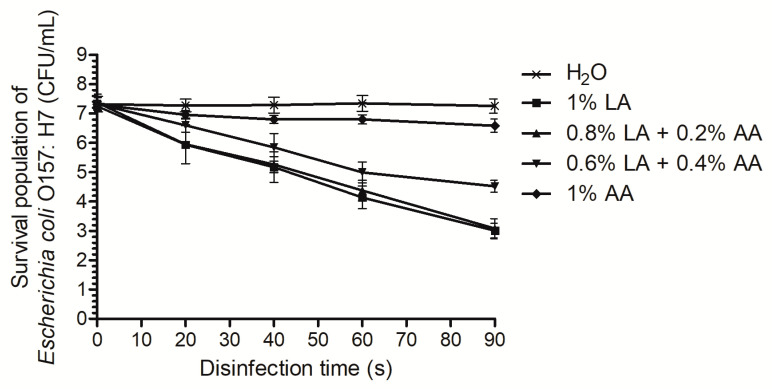
Time-kill curves for different combinations of sanitizers against *Escherichia coli* O157:H7. AA, acetic acid; LA, lactic acid.

Before the fresh produce decontamination and proteomic analyses, it is necessary to determine the time-kill curves under pure culture conditions. We found that 1%AA had the weakest antibacterial effect. After disinfection for 20 s, 1%AA only resulted in a 0.35 log reduction of the *E. coli* O157:H7, and, at the end of the disinfection period (90 s), only a 0.73 log reduction was observed (Figure 1). When the LA was included, the antibacterial effect was improved. In particular, 0.6%LA+0.4%AA reduced the *E. coli* O157:H7 by 0.73 log after 20 s and by 2.81 log at 90 s. When the concentration of LA was increased, 0.8%LA+0.2%AA resulted in a 4.29 log reduction in the *E. coli* O157:H7 within 90 s and showed a similar effect to that of 1% LA. Since long-term disinfection may alter the expression of most proteins, short disinfection periods should be selected for further proteomics analysis [25,28]. Therefore, 0.6%LA+0.4%AA, 0.8%LA+0.2%AA, and 1%LA were selected as the treatment groups for subsequent analyses of the decontamination of fresh produce, and 0.8%LA+0.2%AA and 1%LA for 20 s were selected for a proteomic analysis.

### 3.2. Decontamination Efficacies of Various Combinations against Escherichia coli O157:H7 on Fresh-Cut Baby Spinach and Arugula

**Figure 2:**
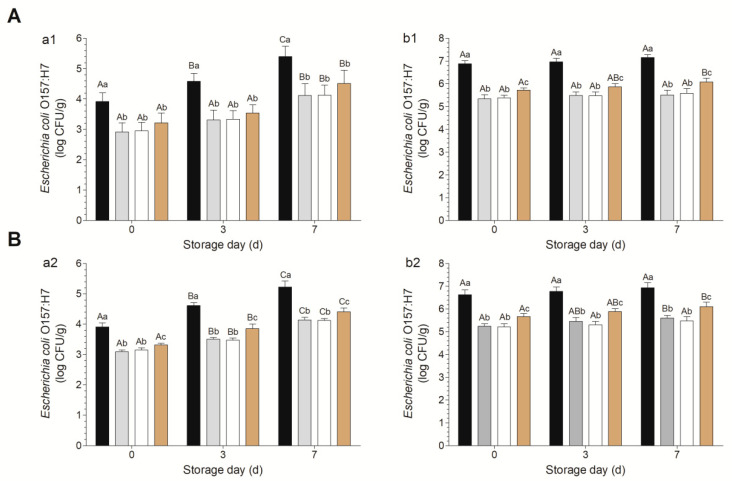
Decontamination effects of different combinations against *Escherichia coli* O157:H7 on fresh-cut spinach (**A**) and arugula (**B**). Panels a and b show results for low and high inoculation levels, respectively. Black, gray, white, and brown bars indicate the results for the control, 1% LA, 0.8% LA plus 0.2% AA, and 0.6% LA plus 0.4% AA, respectively. Note: within the same day, mean values with different lowercase letters are significantly different from each other (*p* < 0.05); within the same treatment, mean values with different capital letters are significantly different from each other (*p* < 0.05).

Owing to the complex and diverse contamination conditions for fresh-cut vegetables [29], two levels of contamination were evaluated in this study, low (10^3^–10^4^ CFU/g) and high (10^6^–10^7^ CFU/g). Under low contamination conditions, the *E. coli* O157:H7 on fresh-cut spinach and arugula increased significantly as the storage time increased (Figure 2a1,a2). The treatment with 1%LA and 0.8%LA+0.2%AA resulted in the lowest counts, with no significant difference between these two groups. From days 3 to 7, the effect of 0.6%LA+0.4%AA was significantly greater than those of the other two groups on fresh-cut arugula (Figure 2a2). For high contamination, the *E. coli* in the control group did not increase significantly from days 0 to 7, consistent with the results of previous studies [14,30], and the effects of 1%LA were similar to those of 0.8%LA+0.2%AA from days 0 to 7, and these two groups showed significantly lower *E. coli* O157:H7 counts than those of the 0.6% LA+0.4%AA group (Figure 2b1,b2). Counts on day 0 and day 7 differed significantly in the 0.6% LA+0.4%AA group but not in the other two groups (Figure 2b1,b2), indicating that 1%LA and 0.8%LA+0.2%AA are more suitable than 0.6%LA+0.4%AA for the decontamination of fresh-cut spinach and arugula with high *E. coli* O157:H7 contamination. In summary, irrespective of the contamination level, 1% LA and 0.8%LA+0.2%AA are recommended, consistent with the results of the time-kill curve analyses.

### 3.3. Effects of Sanitizers on Sensory Characteristics of Fresh-Cut Baby Spinach and Arugula

**Figure 3:**
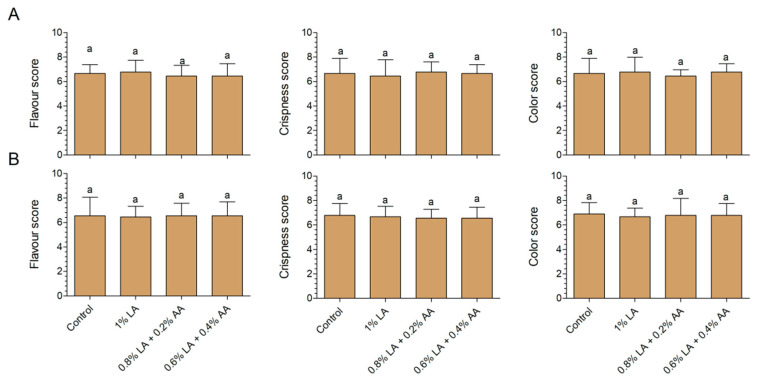
Effects of different combinations of sanitizers on the flavor, crispness, and color of fresh-cut baby spinach (**A**) and arugula (**B**). Different letters above the columns indicate significant differences (*p* < 0.05). AA, acetic acid; LA, lactic acid.

Decontamination will cause damage to the surface of fresh-cut vegetables [14,31]. If the damage is minor, fresh produce will repair itself [32]. If the damage is serious, it will cause a loss of flavor, browning, and water loss [33]. Damage to fresh-cut vegetables is not observed immediately; instead, it appears gradually during storage. In this study, the color, flavor, and crispness at the end of storage (7 d) were evaluated. The three treatments did not negatively affect the sensory qualities compared with those in the control group (Figure 3A,B). However, according to previous studies, AA concentrations exceeding 1% had negative effects on the quality of fresh-cut vegetables. For example, Wang et al. [34] observed browning blots on 1%AA-treated fresh-cut lettuce after storage for 5 d. Vijayakumar and Wolfhall [35] used 6%AA to disinfect fresh-cut lettuce and observed significant reductions in parameters related to appearance, taste, texture, and overall acceptance compared with lettuce treated with lemon juice, apple vinegar, and bleaching powder. When fresh-cut spinach and lettuce were stored for 7 d, the a * value in the AA treatment group was significantly higher than that of the control group [36].

### 3.4. Effects of Sanitizers on the Proteome

Compared with gel-based proteomics, mass spectrometry-based proteomic analyses are now widely used owing to their high-throughput capacity, repeatability, and high success rate for protein identification [37]. In the present study, 15,376 peptides (Table S1) corresponding to 2430 proteins (Table S2) were successfully identified. There were 1755 DEPs (794 upregulated and 961 downregulated) in the LA–CK comparison and 1835 DEPs (761 upregulated and 1074 downregulated) in the AM–CK comparison, indicating that over 50% of the identified proteins were differentially expressed after 20 s of treatment. In AM–LA, 155 DEPs (65 upregulated and 90 downregulated) were found, with substantial overlap between the DEPs in the LA–CK and AM–CK comparisons, which may reflect the slight difference in the LA concentrations. Although the LA concentration differs by 0.2% between the groups, it is possible to achieve similar antibacterial effects via different biological processes.

### 3.5. Venn Diagram Analysis

**Figure 4:**
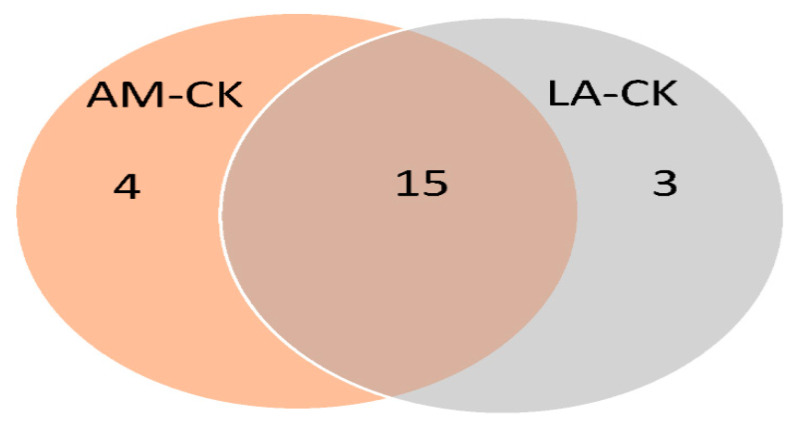
Distribution of biological processes (GO level 3) associated with differentially expressed proteins after treatment with AM and LA. LA: lactic acid; AM: acid mixture, 0.8% LA + 0.2% AA; CK: control.

We evaluated whether the mechanisms underlying the antibacterial effects differ between LA and AM. Therefore, DEPs enrichment for biological processes (BPs), which may be related to the observed antibacterial effects, were evaluated by a GO analysis and a Venn diagram. In total, 15 identical BPs were identified in the two comparisons (Figure 4). This result indicates that, although the difference in the LA concentration between the AM and LA groups is only 0.2%, the BPs affected by the treatment are similar. However, three and four unique BPs, as shown in Figure 4, were identified, indicating that the 0.2% difference in the LA results in changes in the BPs.

### 3.6. Unique Enriched Biological Processes and Network Clustering of LA–CK

The three unique BPs in LA–CK were macromolecule localization, cellular localization, and protein unfolding, associated with 58, 32, and 5 DEPs, respectively. A protein–protein interaction analysis can be used to identify key functional clusters predicted to induce changes in the whole network [38,39,40], and the results indicate protein export (Figure S1A,B), outer membrane, and gram-negative porins (Figure S1A) were involved in the BPs macromolecule and cellular localization.

Protein export is the active transport of proteins from the cytoplasm to the exterior of the cell or to the periplasmic compartment in gram-negative bacteria. In this process, the Sec-dependent pathway, FtsY, and YidC (Table 1) are responsible for transporting newly synthesized proteins into or across the cell membrane [41,42].

**Figure 5:**
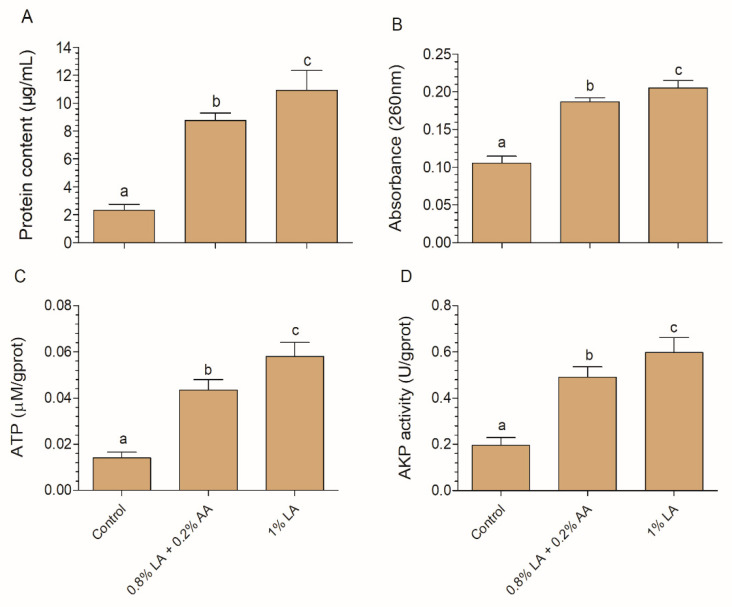
Effects of an acid mixture and lactic acid on protein content (**A**), nucleotide content (**B**), ATP content (**C**), and AKP activity (**D**) in *Escherichia coli* O157:H7. Different letters above the columns indicate significant differences (*p* < 0.05). AA, acetic acid; LA, lactic acid.

Interestingly, another network cluster (i.e., outer membrane and gram-negative porin proteins; Table 1) shows a different location compared to the protein export proteins (typically located on the inner membrane). We hypothesize that 1%LA exerted greater cell membrane damage as compared with 1%AM. Information on the release of the cell constituents reveals the integrity of the cell membrane [43]. Protein, ATP, and nucleotide leakage from intracellular to extracellular spaces can reflect changes in the membrane integrity [31,43,44]. AKP activity in the extracellular environment can reflect the damage extent of the cell membrane. In this work, the leakage of protein, nucleotide, ATP, and AKP was significantly greater for 1% LA than 1% AM (Figure 5), consistent with our hypothesis.

### 3.7. Unique Enriched Biological Processes and Network Clustering of AM–CK

The four unique BPs in AM–CK were taxis, response to stress, catabolic process, and the regulation of molecular function, associated with 18, 233, 203, and 35 DEPs, respectively. According to a protein interaction analysis, the phosphotransferase (PTS) system (Figure S2A) and glycerophospholipid metabolism (Figure S2A), flagellar assembly and bacterial chemotaxis (Figure S2B), and homologous recombination and DNA repair (Figure S3) were involved in the BPs catabolic process, taxis, and response to stress, respectively.

PTS (Table 2) is a distinct mechanism used by bacteria for sugar uptake, where the energy source is phosphoenolpyruvate, which are responsible for the *E. coli* O157:H7 sugar metabolism [45]. Similarly, another study [46] has shown that disodium succinoyl glycyrrhetinate, a derivative of glycyrrhetinic acid, suppresses the sugar metabolism in the cytoplasm at the protein level. The PTS system is multicomponent system that involves enzymes in the plasma membrane and the cytoplasm [47]. Moreover, the PTS system can be activated by acids, antibacterial agents, and salt [48].

Glycerophospholipid metabolism synthesizes the membrane components, and glycerophospholipid drives the formation of the lipid bilayer [49]. In this work, the proteins associated with glycerophospholipid metabolism were located in the cytoplasm and belonged to the major intrinsic protein family (Table 2), which includes transmembrane protein channels, e.g., aquaporins, aquaglyceroporins, and S-aquaporins [50]. According to a previous study, after microorganisms sense a drug, phospholipid synthesis is accelerated for drug transport [51,52], suggesting that the AM applied to the *E. coli* O157:H7 may induce glycerophospholipid metabolism in the cytoplasm.

Homologous recombination and DNA repair (Table 2) are involved in the response to stress and particularly the SOS response. The LexA repressor negatively regulates the SOS genes. Once the pool of LexA decreases, the repression of the SOS genes decreases [53]. In this work, the LexA repressor was upregulated 2.02-fold, indicating that DNA repair was stimulated and that AM may exert more damage against *E. coli* O157:H7 DNA as compared with LA.

Regarding the proteins involved in flagellar assembly and bacterial chemotaxis (Table 2), FliG and FliL are flagellar motor switch proteins and interact with FliM to control the direction of the *E. coli* movement [54]. CheY and CheW are responsible for transmitting the signal obtained from the chemoreceptor to the flagellar rotator in response to an external stimulus [55]. The upregulation of these proteins (Table 2) indicated that AM may exert greater chemical stress than LA. In a previous study [56], oil isolated from fingered citron promoted chemotaxis and flagella assembly in *Listeria monocytogenes* at the transcriptomic level.

## 4. Conclusions

The effects of LA and AM on *E. coli* O157:H7 on fresh-cut produce as well as the mode of action at a proteome level were evaluated. A produce decontamination experiment showed that LA and AM have similar decontamination efficacies against the *E. coli* O157:H7 on fresh-cut spinach and arugula, consistent with the results of the time-kill curves. In addition, the AM and LA did not negatively affect the sensory characteristics of the fresh-cut produce after storage. Accordingly, AM is a cheaper alternative to LA for the decontamination of fresh-cut produce. At the proteome level, LA and AM affected distinct biological processes. In particular, macromolecule localization, cellular localization, and protein unfolding were uniquely affected by LA, and taxis, response to stress, catabolic process, and the regulation of molecular function were uniquely changed after the treatment with AM. Further network clustering and cellular component analyses showed that membrane damage that may be induced by LA is greater than that induced by AM. Additionally, AM may have a stronger inhibitory effect on the biological processes in the cytoplasm, including DNA repair, bacterial chemotaxis, sucrose metabolism, and glycerophospholipid metabolism. Cell membrane permeability experiments confirmed that LA has a stronger damaging effect on cell membranes than AM. In addition, *E. coli* O157:H7 is a hazard for fresh meat, and lactic acid is used in various countries to decontaminate fresh meat. Whether the decontamination effect of AM is consistent with that of LA should also be evaluated in future studies. In addition, the relationship between decontamination and ecological changes should be further evaluated, including in-depth 16S rRNA and metatranscriptomic studies.

## Figures and Tables

**Table 1:** Related differentially expressed proteins in network clusters of LA–CK.

UniProt Accession Number	Gene ID	Protein Name	Fold Change
Protein export			
P0AG88	secB	Protein-export protein SecB	2.35
P0AGA1	secG	Protein-export membrane protein SecG	2.06
P0AGA4	secY	Protein translocase subunit SecY	0.53
P0AG91	secD	Protein translocase subunit SecD	0.65
P0AG95	secF	Protein translocase subunit SecF	1.24
P65625	yidC	Membrane protein insertase YidC	0.43
P0AGD9	ffh	Signal recognition particle protein	0.38
Outer membrane and gram-negative porins			
P61318	lolA	Outer-membrane lipoprotein carrier protein	0.82
P0ADC5	lolC	Lipoprotein-releasing system transmembrane protein LolC	0.37
P0ABV8	tolR	Tol-Pal system protein TolR	2.07
P0ABV0	tolQ	Tol-Pal system protein TolQ	0.46
Q8X8E2	lolE	Lipoprotein-releasing system transmembrane protein	0.45
P0AC04	bamD	Outer membrane protein assembly factor BamD	2.45
P0AEU9	skp	Chaperone protein Skp	2.25
P0A9V3	lptB	Lipopolysaccharide export system ATP-binding protein LptB	1.42

LA: lactic acid; CK: control.

**Table 2:** Related differentially expressed proteins in network clusters of AM–CK.

UniProt Accession Number	Gene ID	Protein Name	Fold Change
Starch and sucrose metabolism, and phosphotransferase system			
Q8XDG9	mtlD	Mannitol-1-phosphate 5-dehydrogenase	1.27
Q8X677	manA	Mannose-6-phosphate isomerase	0.27
Q8XE22	pfkB	Phosphofructokinase	0.59
A0A0H3JCR2	pgm	Phosphoglucomutase	0.35
P0AB72	fbaA	Fructose-bisphosphate aldolase class 2	0.57
P62709	gpmA	2,3-bisphosphoglycerate-dependent phosphoglycerate mutase	2.34
P0A6V9	glk	Glucokinase	0.60
Q8XCE1	treC	Trehalose-6-P hydrolase	0.38
Q8X710	malQ	4-alpha-glucanotransferase	0.37
Q8X6Y1	glgP	Alpha-1,4 glucan phosphorylase	0.41
Q8X6X8	glgX	Glycogen debranching enzyme	0.34
Glycerophospholipid metabolism and major intrinsic proteins			
P0A997	glpC	Anaerobic glycerol-3-phosphate dehydrogenase subunit C	0.59
P0A6F4	glpK	Glycerol kinase	0.55
P0A9C1	glpA	Anaerobic glycerol-3-phosphate dehydrogenase subunit A	0.69
A0A0H3JI74	glpD	Glycerol-3-phosphate dehydrogenase	0.60
P0A6S9	gpsA	Glycerol-3-phosphate dehydrogenase [NAD(P)+]	0.38
Flagellar assembly and bacterial chemotaxis			
P0A966	cheW	Chemotaxis protein CheW	2.83
P0AE68	cheY	Chemotaxis protein CheY	2.80
Q8XCF9	cheB	Protein-glutamate methylesterase/protein-glutamine glutaminase	0.60
P0ABZ3	fliG	Flagellar motor switch protein FliG	1.67
P0ABY0	fliL	Flagellar protein FliL	2.00
Homologous recombination and DNA repair			
Q8X8H1	polA	DNA polymerase I	0.78
P0A7G8	recA	Protein RecA	1.41
P0A7H2	recF	DNA replication and repair protein RecF	0.67
Q8XDN4	mutL	DNA mismatch repair protein MutL	0.40
Q8X8P5	uvrD	DNA helicase	0.58
P0A811	ruvA	Holliday junction ATP-dependent DNA helicase RuvA	0.69
Q8X5H9	ftsK	DNA translocase FtsK	0.21
P0A7C4	lexA	LexA repressor	2.07

AM: acid mixture; CK: control.

## Data Availability

The data presented in this study are available on request from the corresponding author (J.W.), upon reasonable request.

## Supplementary Materials

The following are available online at https://www.mdpi.com/article/10.3390/foods10102406/s1, Figure S1: Protein–protein interaction network for differentially expressed proteins involved in macromolecule localization (A) and cellular localization (B). Yellow points in A indicate the network cluster related to cell outer membrane and gram-negative porin; red points in A and B indicate the network cluster related to protein export, Figure S2: Protein–protein interaction network for differentially expressed proteins involved in catabolic process (A) and taxis (B). Blue points in A indicate the network cluster related to starch and sucrose metabolism, and phosphotransferase system; red points in A indicate the network cluster related to glycerophospholipid metabolism and major intrinsic protein; red points in B indicate the network cluster related to flagellar assembly and bacterial chemotaxis, Figure S3: Protein–protein interaction network for differentially expressed proteins involved in the response to stress. Red points indicate the network cluster related to homologous recombination and DNA repair, Table S1: Peptide information for identified proteins, Table S2: Information on the identified proteins.
