# Supplementary figures and images for: Use of Acetic Acid to Partially Replace Lactic Acid for Decontamination against Escherichia coli O157:H7 in Fresh Produce and Mechanism of Action
Source: Foods. 2021 Oct 11;10(10):2406. doi: 10.3390/foods10102406 (PMC8535275; doi:10.3390/foods10102406)

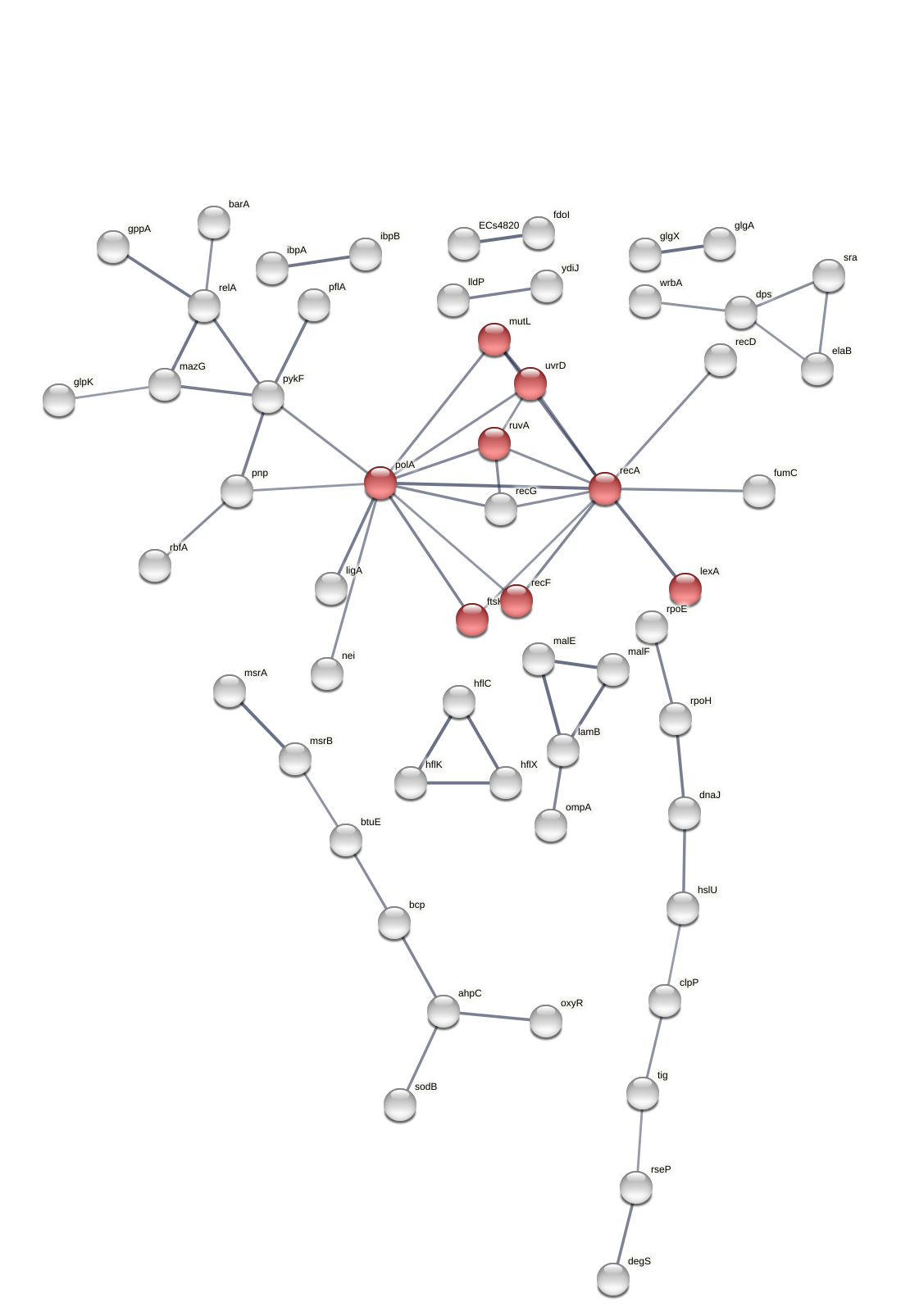

Supplement: Supplementary file 1 [file foods-10-02406-s001.zip › Figure S3.png]
